# Supplementary material for: Quantitative live cell imaging reveals influenza virus manipulation of Rab11A transport through reduced dynein association
Source: Nat Commun. 2020 Jan 7;11:23. doi: 10.1038/s41467-019-13838-3 (PMC6946661; doi:10.1038/s41467-019-13838-3)
Supplement: Supplementary file 1 — Supplementary Information [file 41467_2019_13838_MOESM1_ESM.pdf]

Non-deconvolved

Bead no.	FWHM-x	FWHM-y	FWHM-z
2	0.47	0.48	1.53
3	0.4	0.42	1.53
5	0.34	0.36	1.53
10	0.4	0.41	1.43
11	0.37	0.41	1.4
12	0.38	0.4	1.78
15	0.41	0.37	1.62
16	0.4	0.41	1.22
17	0.38	0.41	1.28
18	0.4	0.4	1.31
19	0.38	0.4	1.35
20			
21	0.4	0.41	1.14
22	0.37	0.38	1.09
23	0.39	0.4	1.36
24	0.38	0.39	1.48
25	0.45	0.45	1.28
27			
28	0.39	0.39	1.38
29	0.4	0.4	1.32
30	0.43	0.41	1.47
31	0.38	0.38	1.21
33	0.41	0.4	1.29
34	0.4	0.42	1.27
35			
36	0.4	0.41	1.25
41	0.4	0.37	1.39
42	0.4	0.38	1.32
44	0.37	0.37	0.97
avg	0.396154	0.401154	1.353846
SD	0.02578	0.02535	0.170084

Deconvolved

Bead no.	FWHM-x	FWHM-y	FWHM-z
2	0.25	0.3	0.32
4	0.25	0.34	0.4
6	0.26	0.34	0.35
7	0.25	0.28	0.34
8	0.3	0.27	0.34
10	0.26	0.35	0.34
11	0.29	0.29	0.39
14	0.24	0.27	0.35
16	0.24	0.29	0.34
17	0.22	0.33	0.34
18	0.32	0.33	0.44
19	0.24	0.28	0.29
20	0.28	0.3	0.35
22	0.27	0.29	0.33
23	0.26	0.34	0.41
24	0.25	0.33	0.36
25	0.32	0.26	0.36
28	0.29	0.31	0.37
29	0.3	0.34	0.38
30			
31	0.24	0.3	0.35
32			
33	0.3	0.32	0.35
34	0.3	0.33	0.36
35	0.23	0.31	0.37
36	0.26	0.34	0.33
37	0.23	0.3	0.35
38	0.31	0.27	0.41
avg	0.267692	0.308077	0.358462
SD	0.029841	0.027132	0.031836

Supplementary Table 1: Measured FWHM values for 100-nm fluorescent beads. Measured values for FWHM (μm) of beads described in Supplementary Figure 1 before and after deconvolution. The empty rows denote beads that were ignored because multiple beads were found in close proximity, which hindered accurate measurement of FWHM. Calculated average (avg) and standard deviation (SD) are also denoted for the measured samples.

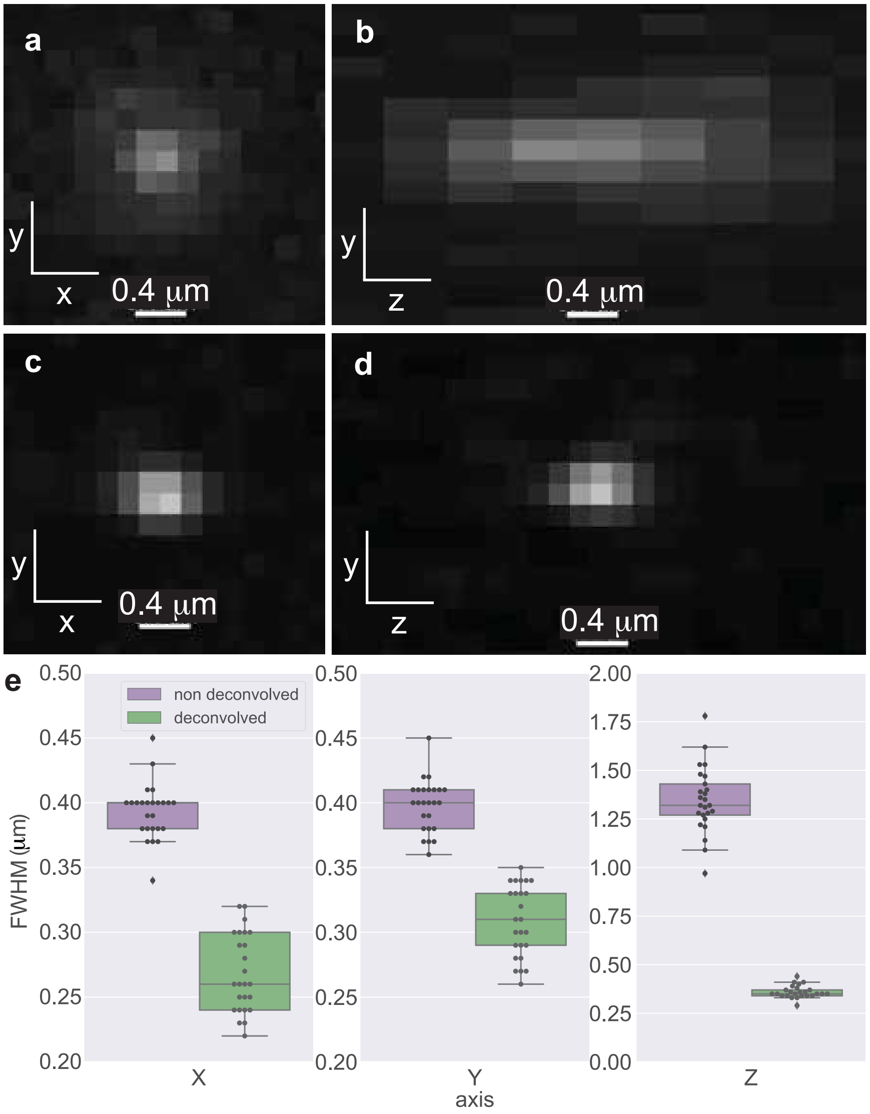

Supplementary Figure 1: Characterization of diSPIM using 100-nm green fluorescent beads. Green fluorescent beads (see Materials and Methods) were embedded in 0.2% agarose gel and imaged under the same conditions as for cells. 10 successive volumes were obtained. The images were analyzed in Imaris to identify spots, both before and after fusion, and the subvolumes encompassing the spots were saved as individual image stacks. **a**, X-Y and **b**, Y-Z maximum projections of a bead subvolume before deconvolution. **c**, X-Y and **d**, Y-Z maximum projections of a bead subvolume after deconvolution. **e**, Distribution of full width at half-maximum (FWHM) measured along each axis before (n=26 beads) and after (n = 26 beads) deconvolution, displayed as box and whisker plots using statistical routines built into Python's Seaborn package. Boxes represent the quartiles of the dataset, while whiskers represent 1.5 times inter-quartile range. All individual measurements are also overlaid. Source data are shown in Supplementary Table 2.

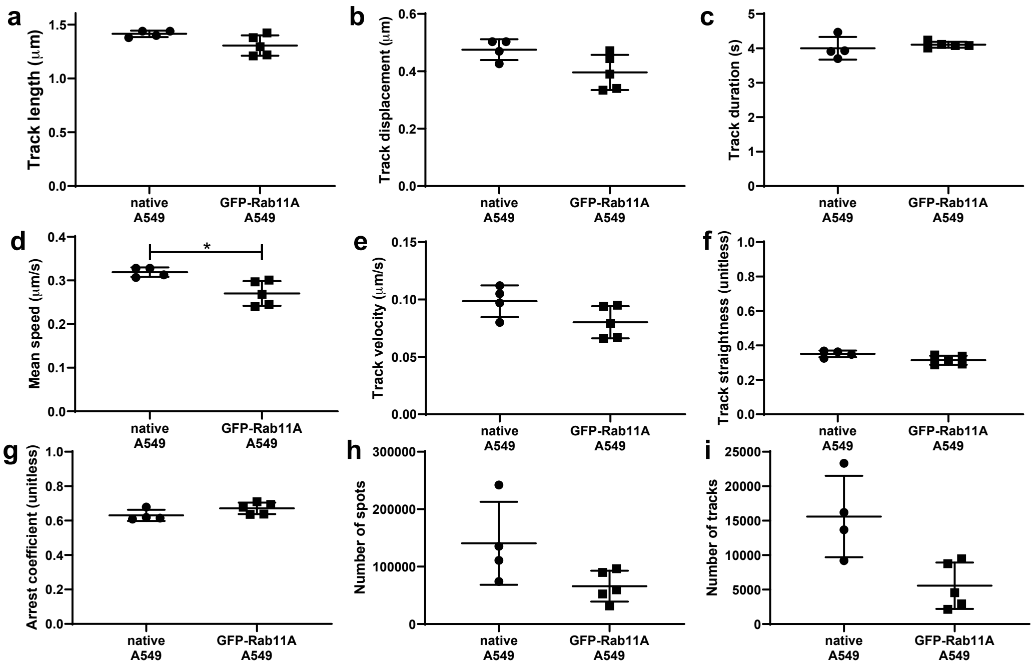

Supplementary Figure 2: Comparison of motion parameters for transferrin between native A549 cells and GFP-Rab11A A549 cells. Live-imaging of cells incubated with Alexa 568-tagged transferrin, a known cargo of Rab11A-RE, was performed in order to quantify the effect of stable transfection on GFP-tagged Rab11A on A549 cells. Cells were incubated with 25 µg/ml of labeled transferrin for 30 minutes in Fluorobrite Dulbecco's Modified Eagle Medium (DMEM) supplemented with 1% bovine serum albumin according to manufacturer protocols. The transferrin was replaced with imaging media (Fluorobrite DMEM) and cells were imaged with the diSPIM. Image processing and analysis of transferrin puncta motion was performed as described in Methods. Each data point represents an individual analyzed cell (native A549: n=4 cells, GFP-Rab11A A549: n=5 cells) using CF_{50} for the corresponding motion parameters: **a**, track length, **b**, track displacement, **c**, track duration, **d**, track mean speed, **e**, track velocity, **f**, track straightness, **g**, track arrest coefficient, **h**, total number of spots, and **i**, total number of tracks. Mean ± SD is shown per group (* implies $p<0.05$, comparisons performed using non-parametric Mann-Whitney U test). Source data are provided as a Source Data file.

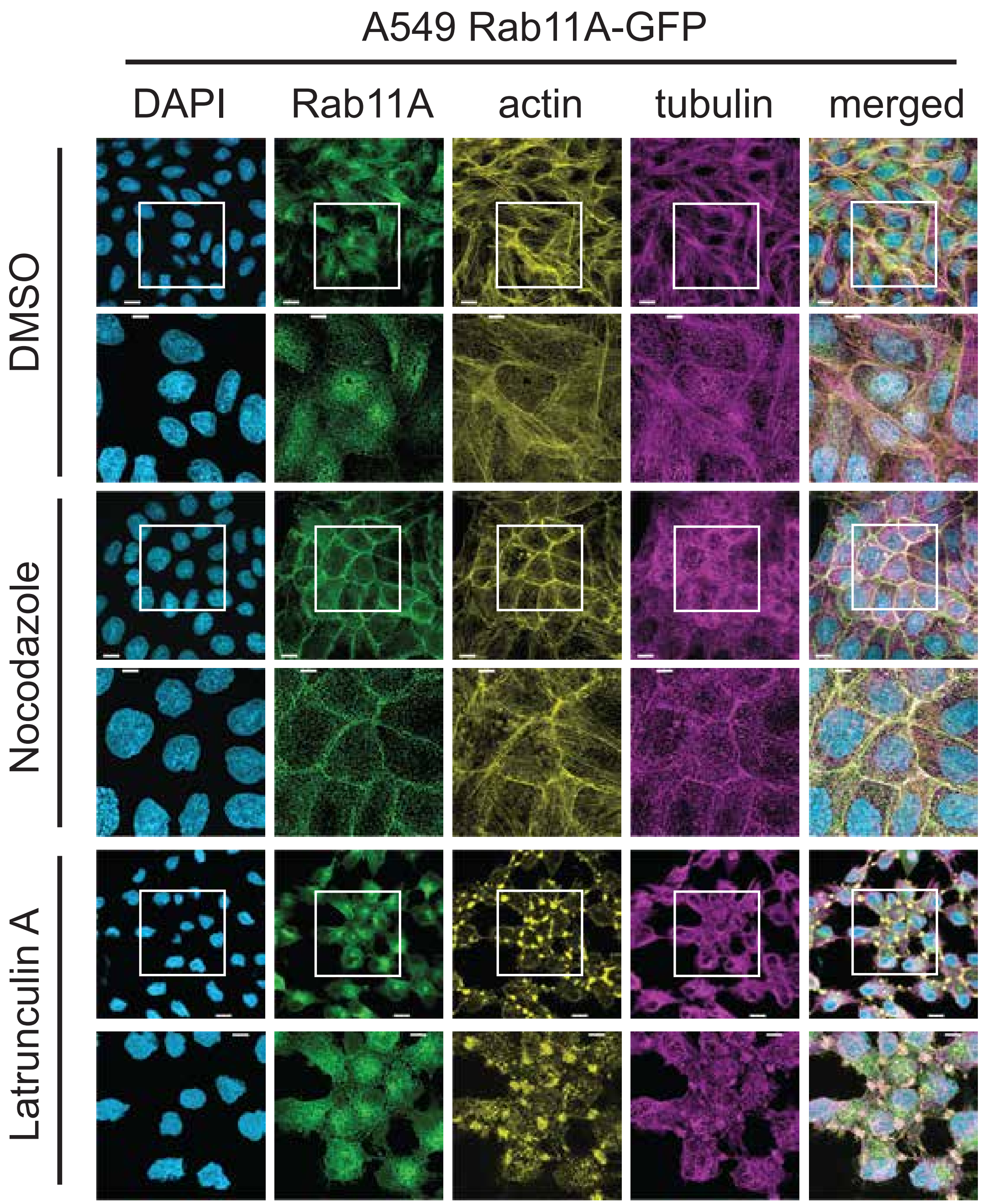

Supplementary Figure 3: Widefiled imaging confirms cytoskeletal filament disruption with drug treatment in GFP-Rab11A expressing cells. A549 cells stably expressing GFP-Rab11A were treated with nocodazole (16 µM), latrunculin A (1 µM) or DMSO (control) and were imaged on the diSPIM 4 h post treatment. After imaging, the cells were fixed and stained with antibodies corresponding to microtubules or actin, to confirm the depolymerization of each microfilament type under its drug treatment. The various cellular components labeled are denoted on the top, while the treatments are labeled on the left side. Each drug treatment is shown in a wide field view and then a single cell is shown to clearly demonstrate the destruction of the cytoskeletal protein. Images show complete depolymerization of microtubules (rows 3, 4) and actin network (rows 5, 6) compared to control (DMSO, rows 1, 2). DAPI (4′, 6-diamidino-2-phenylindole, shown in blue) is used to demarcate nuclei. Scale bars are 10 µm (5 µm for inset images).

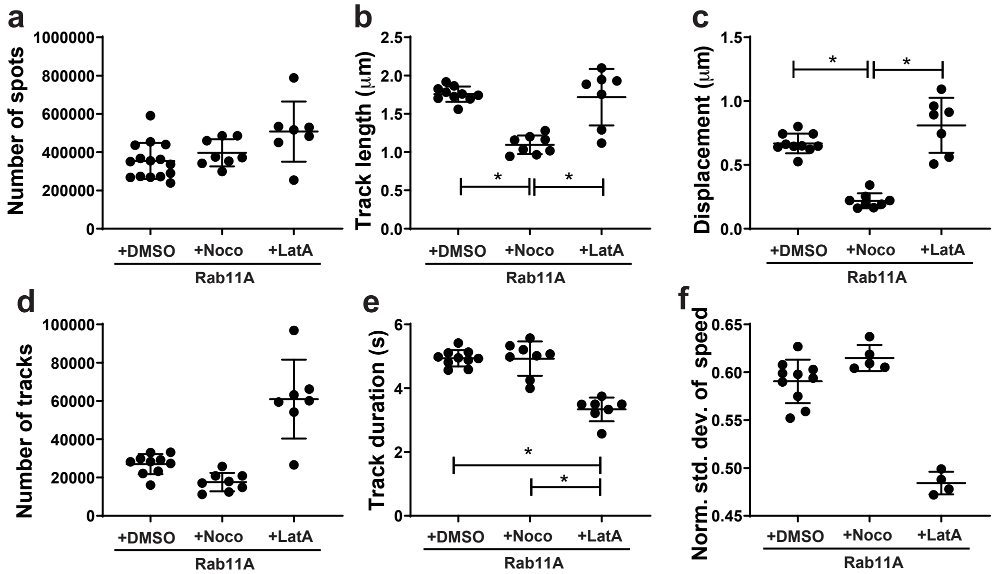

Supplementary Figure 4: Impact of cytoskeletal depolymerizing drugs on the transport of Rab11A. Here we present additional motion parameters that complement the data presented in Figure 3. Each data point represents an individual analyzed cell (DMSO: n=10 cells, nocodazole: n=8 cells, latrunculin A: n=7 cells) using CF_{50} for the corresponding motion parameters: **a**, number of spots, **b**, track length, **c**, track displacement, **d**, number of tracks, **e**, track durations, and **f**, normalized standard deviation of track speed from multiple cells. Image processing and analysis was performed as described in Methods Section. Mean ± SD is shown per group (* implies $p<0.05$, comparisons performed using 1-way ANOVA with Tukey's multiple tests). In the case of latrunculin A treated cells, the number of spots observed was similar to other treatments, but the number of tracks was disproportionately high. The apparently high number of tracks may be due to the large number of possible fission and fusion events, which generate new tracks within the Imaris software. Source data are provided as a Source Data file.

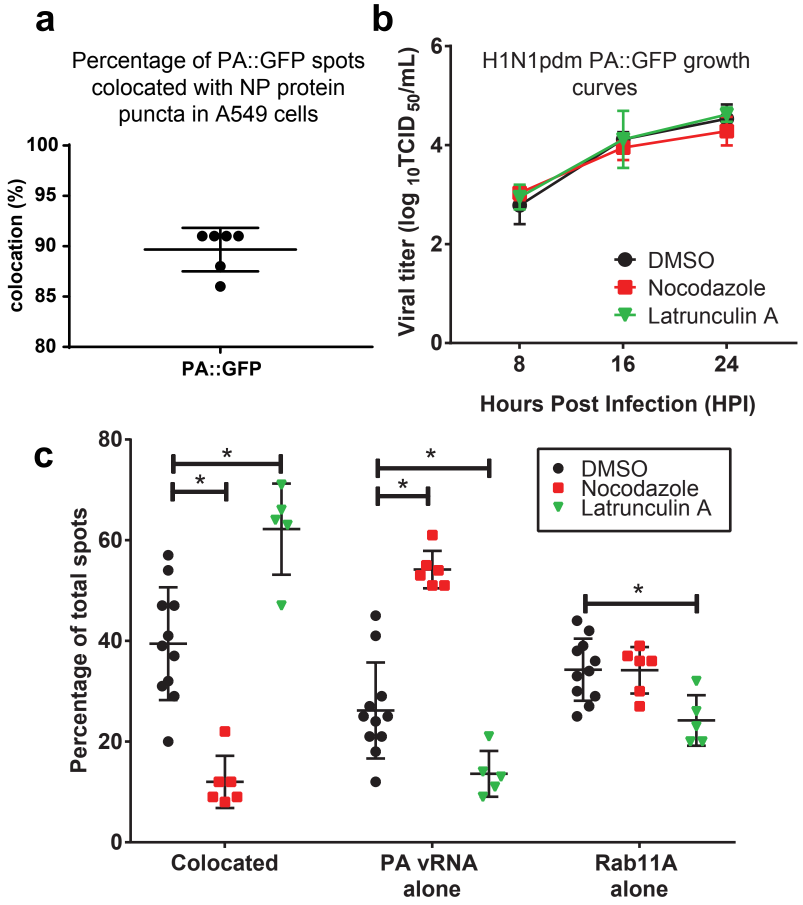

Supplementary Figure 5: Characterization of H1N1pdm PA::GFP virus for live cell imaging. a, Colocalization of PA::GFP and NP was performed in A549 cells infected with H1N1pdm PA::GFP at a MOI = 1. Cells were fixed at 16 hours post infection (hpi) and stained for IAV NP protein. Quantification of the cytoplasmic PA::GFP and anti-NP puncta colocalization was performed on deconvolved images. Each data point represents the percentage of diffraction limited spots containing both PA::GFP and NP from an individual cell (n=6 cells). Average and standard deviation are shown. On average, ~90% of PA::GFP cytoplasmic foci collocated with NP spots, which shows that PA::GFP is a good surrogate for imaging IAV vRNP dynamics. **b**, Replication of H1N1pdm PA::GFP in A549 cells. Infected cells were treated with drugs 4 hpi and maintained in media with drugs for up to 24 hpi. Cellular supernatants were collected at 8, 16 and 24 hpi and viral titration at each timepoint was determined. Each measurement used 3 technical replicates (n = 3 replicates). **c**, Colocalization of vRNP and GFP-Rab11A. A549 GFP-Rab11A cells were infected with WSN virus (H1N1 A/WSN/33), treated with cytoskeletal drugs 4 hpi and fixed at 8 hpi. RNA-FISH was performed to identify locations of PA vRNP segment and images were subjected to colocalization analysis (see methods). Each data point represents spots from within individual cells (DMSO: n=11 cells, nocodazole: n=6 cells, latrunculin A: n=5 cells). Comparisons were performed using two-way ANOVA with Tukey's multiple comparison tests (* denotes $p<0.05$). PA vRNP and Rab11A colocalization reduced significantly under microtubule depolymerization (nocodazole, filled red squares) compared to control (DMSO, black filled circles) and correspondingly the number of non-colocated PA-vRNP increased (column 2). On the other hand, actin depolymerization resulted in an increase in Rab11A-colocalization (green filled triangles) compared to DMSO treatment. Mean ± SD are shown per group. Source data are provided as a Source Data file.

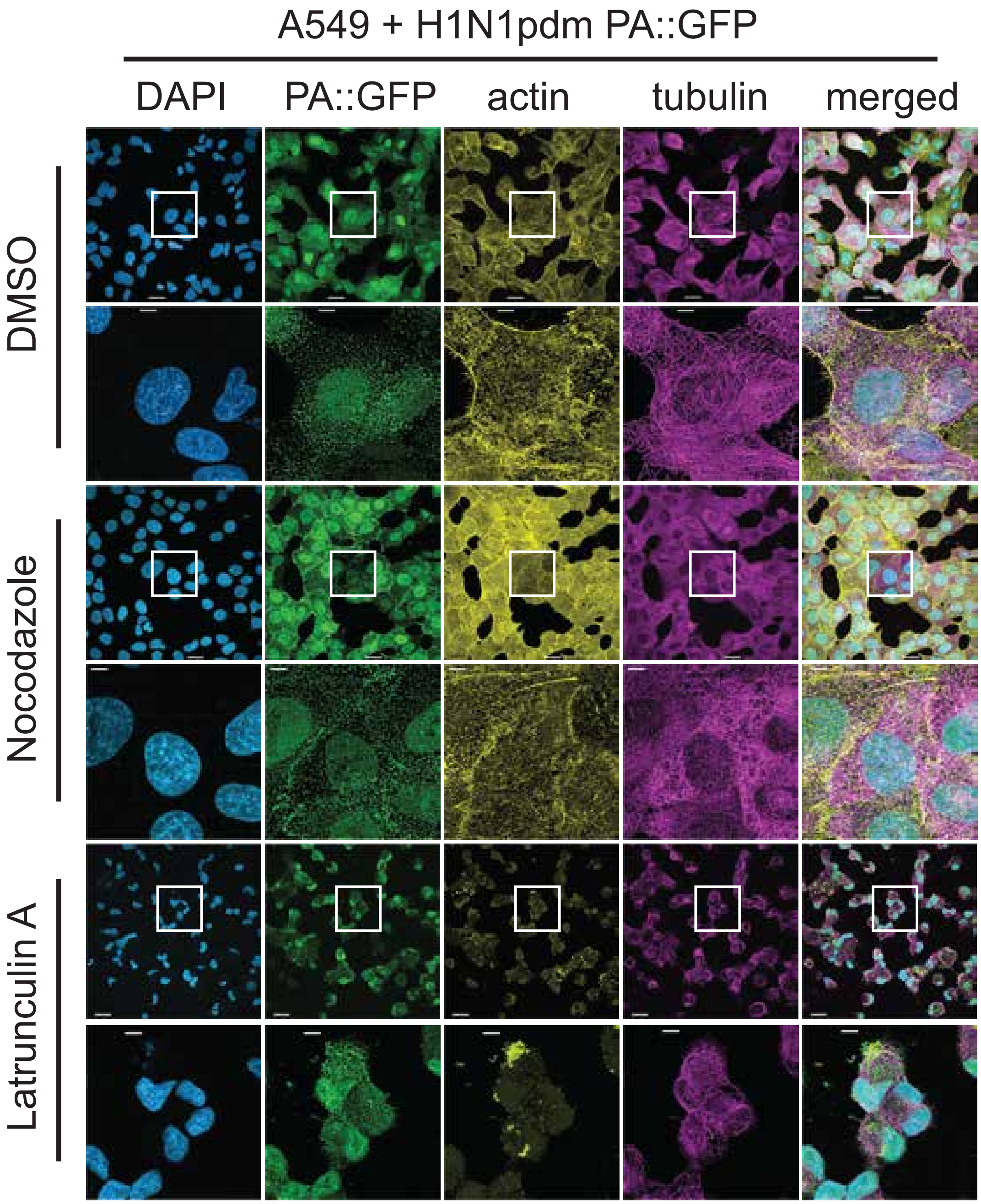

Supplementary Figure 6: Widefield microscopy confirms cytoskeletal filament disruption with drug treatment in infected cells. A549 were infected with H1N1pdm PA::GFP virus, treated with cytoskeletal drugs 4 hpi and imaged live at 16 hpi. Post-imaging, the cells were fixed and immunofluorescence performed with tubulin and actin antibodies. Each drug treatment is shown in a wide field view and then a single cell is shown to clearly demonstrate the destruction of the cytoskeletal protein. Images show complete depolymerization of microtubules (rows 3, 4) and actin network (rows 5, 6) compared to control (DMSO, rows 1, 2). DAPI (blue) is used to demarcate nuclei. Scale bars are 20 μm (5 μm for inset images).

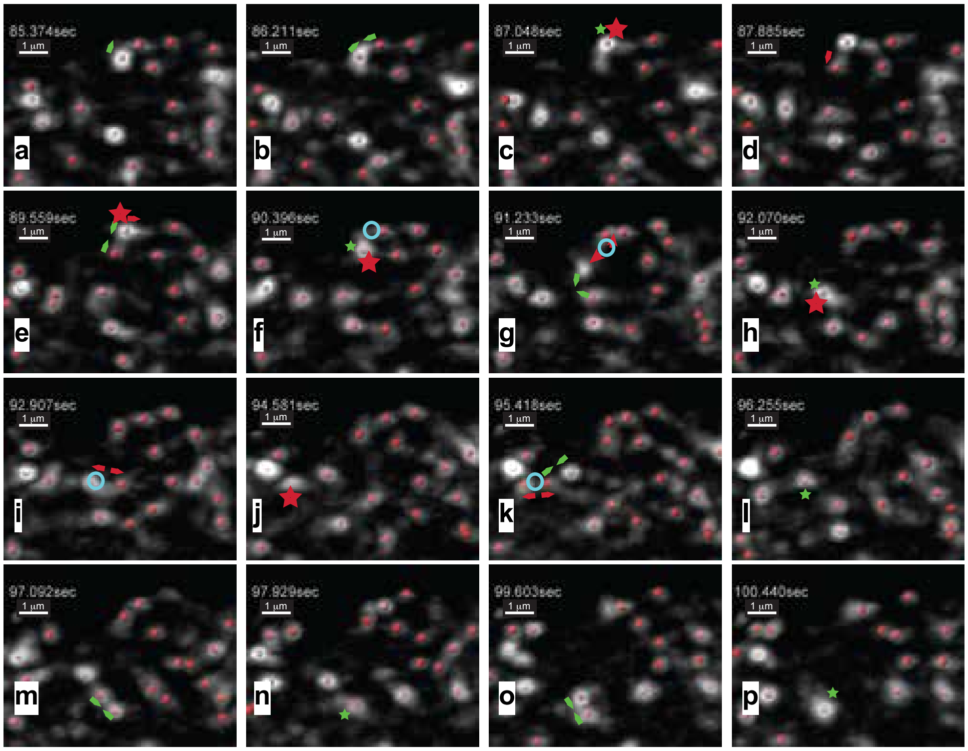

Supplementary Figure 7: Dynamic nature of influenza vRNP movement captured by colocalization, merging and splitting of PA::GFP puncta. Snapshots of interaction between PA::GFP puncta from a representative dataset are shown here. A merging interaction is shown using green arrows and the newly merged spot is denoted with a filled green star (frames c, f, h, l, n and p). A splitting instance is shown using red arrows, and the spot about to split is denoted by a red star in the previous frame (frames c, e, f, h and j). A blue circle represents the former location of a spot that split into two (frames f, g, i, and k). Sometimes the same PA::GFP spot is labeled with a green and red star to denote that it was formed by a merging of spots and will split in the near future (frames c, f and h). This "temporary colocalization" interaction between PA::GFP spots is observed frequently in our influenza imaging datasets and probably leads to the correct assembly of the influenza genome via vRNP-vRNP interactions. Timestamps are measured from the beginning of the dataset.

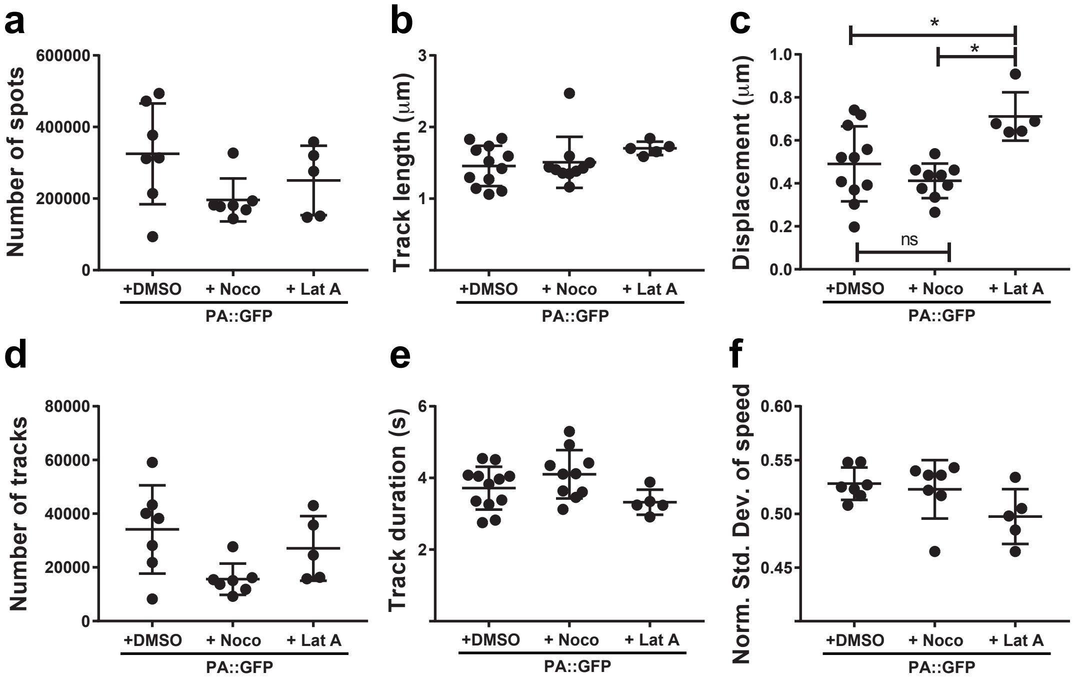

Supplementary Figure 8: Impact of cytoskeletal depolymerizing drugs on the transport of PA::GFP. Here we present additional motion parameters that complement the data presented in Figure 4. Each data point represents an individual analyzed cell (DMSO: n=10 cells, nocodazole: n=9 cells, latrunculin A: n=5 cells) using CF50 of the corresponding motion parameters: **a**, number of spots, **b**, track length, **c**, track displacement, **d**, number of tracks, **e**, track durations, and **f**, normalized standard deviation of track speed from multiple cells. Image processing and analysis was performed as described in Methods. Mean ± SD is shown per group (* implies $p<0.05$, comparisons performed using 1-way ANOVA with Tukey's multiple tests). Source data are provided as a Source Data file.

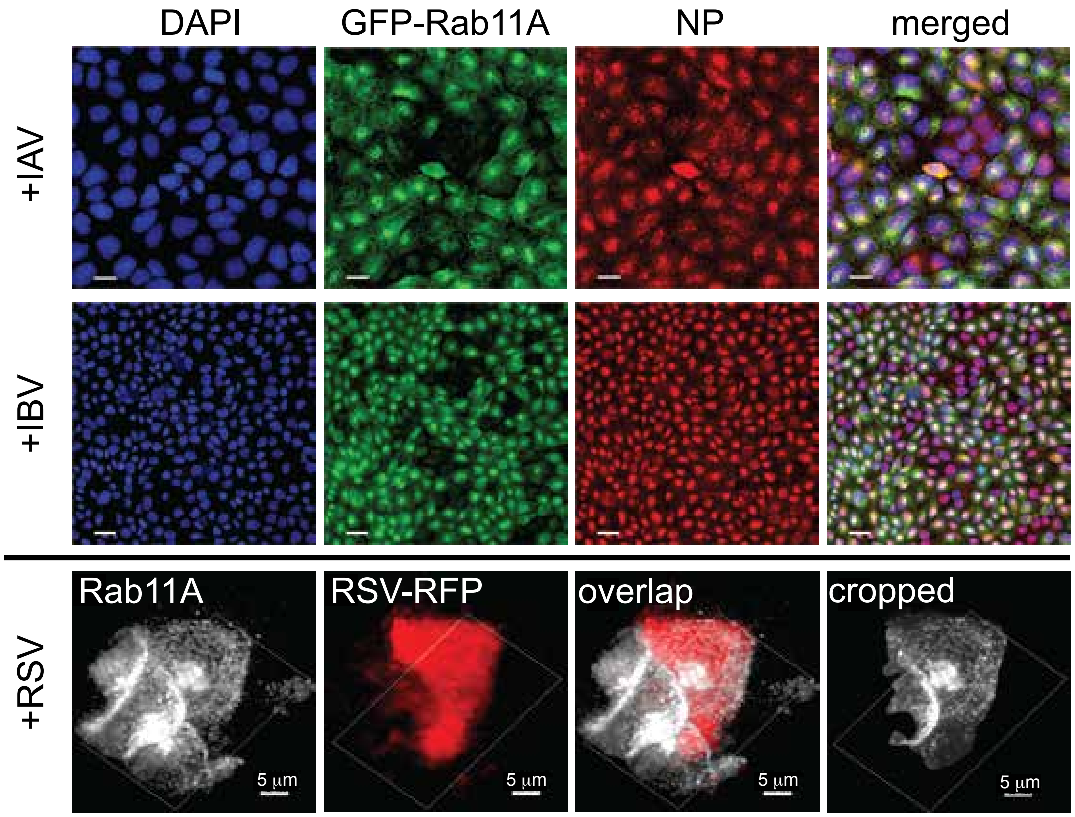

Supplementary Figure 9: Confirmation of virus infection in A549 GFP-Rab11A cells. Row 1 and 2: Widefield images of cells infected with IAV (A/WSN/33) and IBV (B/Texas/02/2013) at a high MOI (= 3), to confirm that a majority of the cells were infected. Post live imaging on diSPIM, cells were fixed and immunostained with IAV and IBV NP antibodies respectively (column 3, red). Images show almost a 100% infection coverage, which confirms that any cell analyzed on average has a very high likelihood of being infected. Nuclei are stained with DAPI (blue). Scale bars are 15 µm (row 1, WSN) and 30 µm (row 2, IBV). Row 3: dSPIM image of A549 GFP-Rab11A cells infected with recombinant RSV (expressing a reporter red fluorescent protein [RFP]) at 0.1 pfu per cell. RFP positive cells were identified and live imaging performed on diSPIM. RFP fluorescence was used to isolate data exclusively from infected cells for further analysis. Scale bars as shown in the figure.

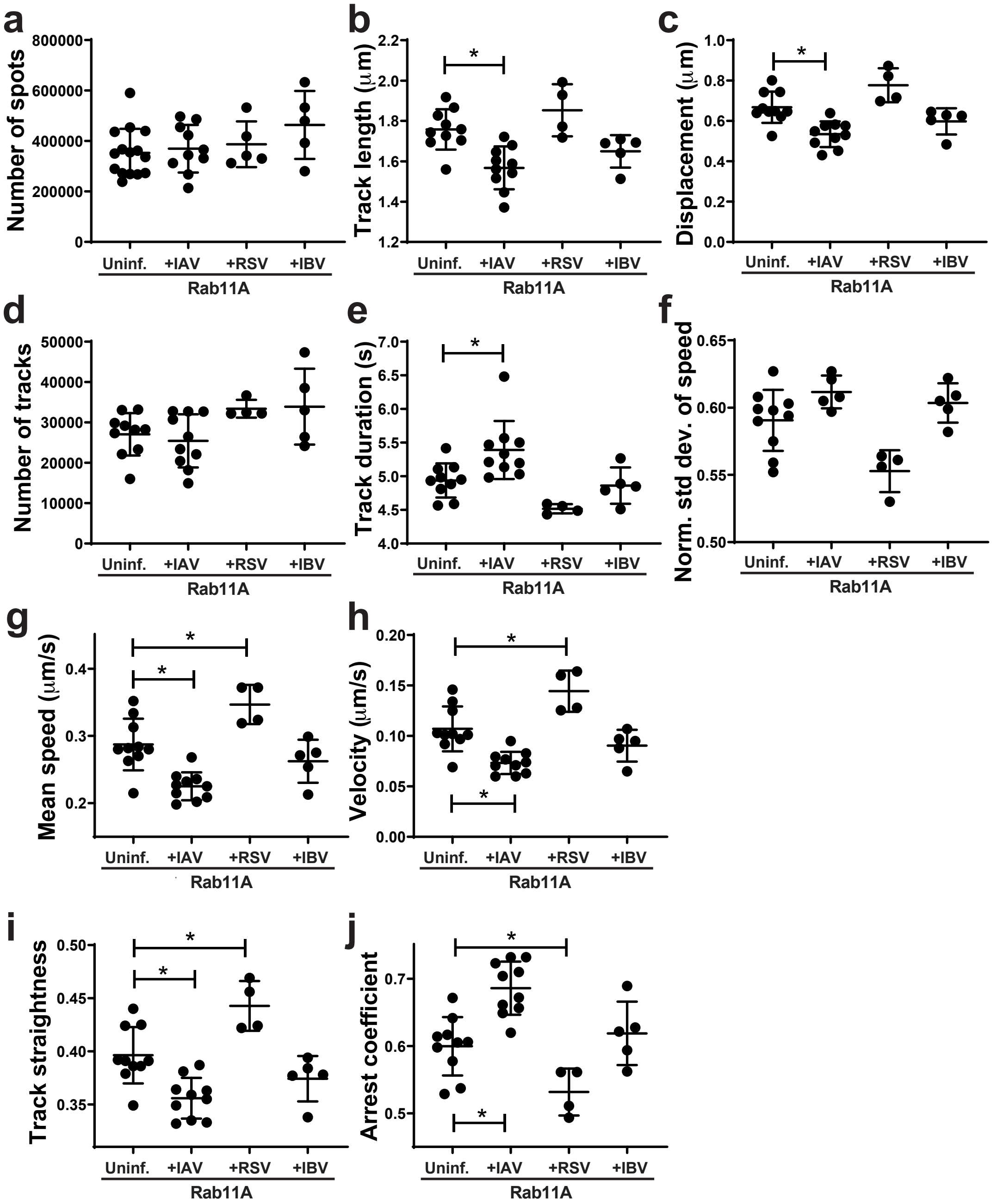

Supplementary Figure 10: Impact of viral infection on the transport of GFP-Rab11A. Here we present additional motion parameters that complement the data presented in Figure 5 and include all parameters resulting from influenza B virus (IBV) infection. Each data point represents an individual analyzed cell (uninfected: n=10 cells, WSN: n=10 cells, RSV: n=4 cells, IBV: n=5 cells) using CF_{50} of the corresponding motion parameters: **a**, number of spots, **b**, track length, **c**, track displacement, **d**, number of tracks, **e**, track durations, **f**, normalized standard deviation of track speed, **g**, track mean speed, **h**, track mean velocity, **i**, track straightness, and **j**, track arrest coefficients from multiple cells. Image processing and analysis was performed as described in Methods. Mean ± SD is shown per group (* implies p<0.05, comparisons performed using 1-way ANOVA with Tukey's multiple tests). Source data are provided as a Source Data file.

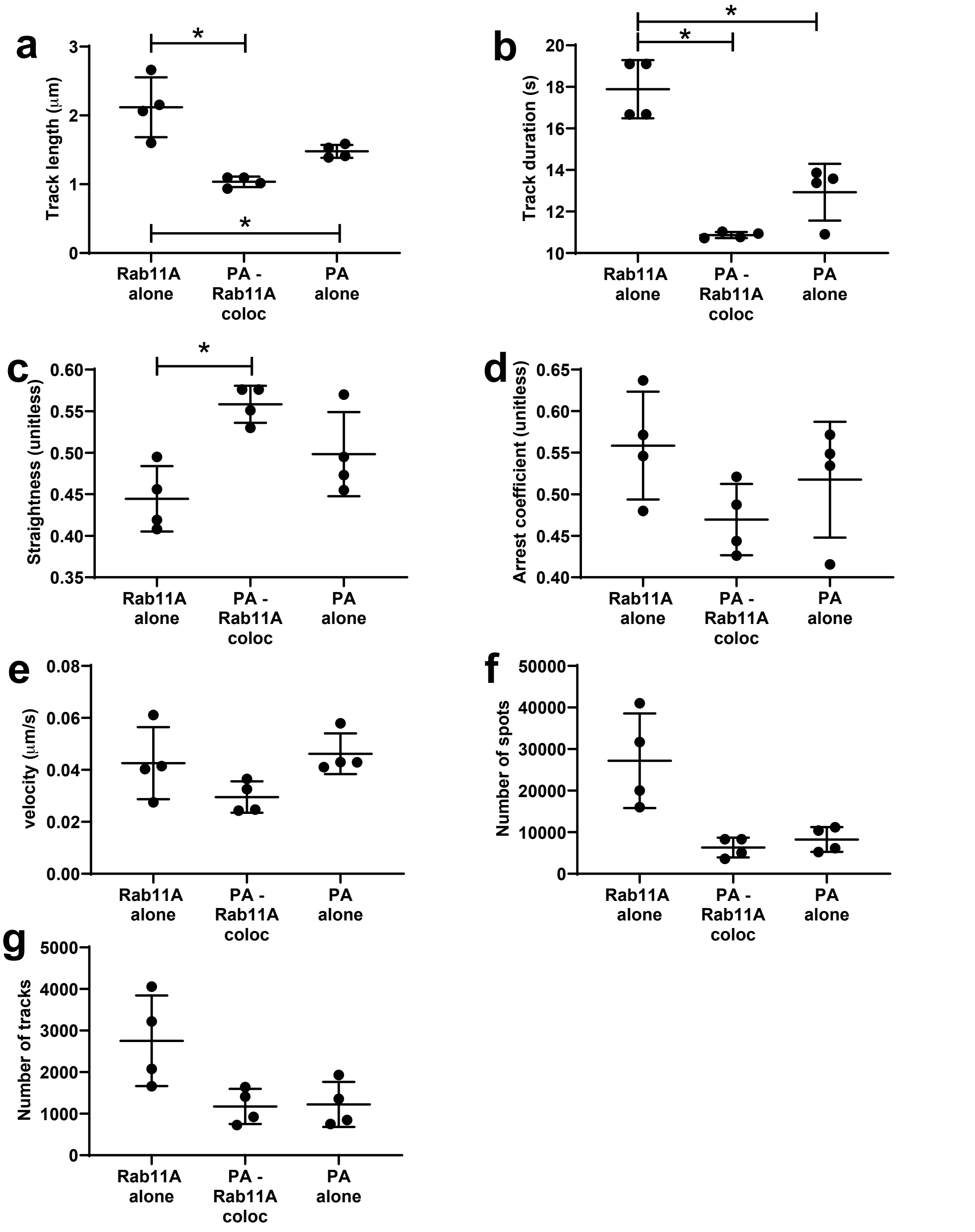

Supplementary Figure 11: Additional motion parameters for two-color imaging datasets. GFP-Rab11A cells were infected with IAV PA::mRuby, imaged at 16 hpi and the image processing and analysis was performed as described in Materials and Methods. Here, GFP-Rab11A spots were analyzed separately as spots either colocalized (PA-Rab11A coloc) or non-colocalized (Rab11A alone) with PA::mRuby. Similarly, PA::mRuby were analyzed as spots non-colocated with GFP-Rab11A (PA::mRuby alone). Each data point represents an individual analyzed cell (n=4 cells) using CF_{50} of the corresponding motion parameters: **a**, track length, **b**, track durations, **c**, track straightness, **d**, track arrest coefficients, **e**, track mean velocity, **f**, total number of spots, and **g**, number of tracks from multiple cells. Mean ± SD is shown per group (* implies $p<0.05$, comparisons performed using 1-way ANOVA with Tukey's multiple tests). Source data are provided as a Source Data file.

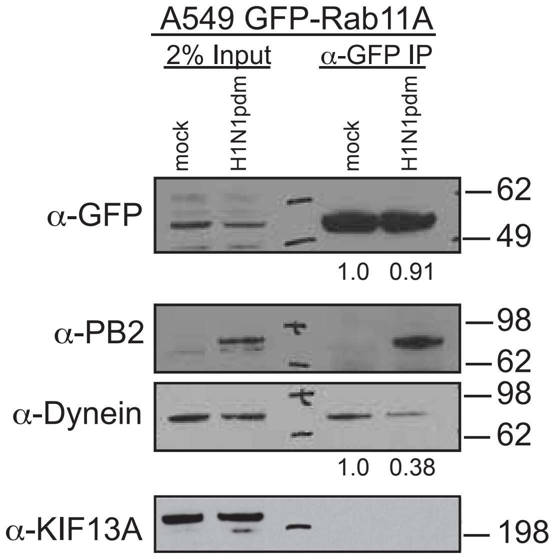

Supplementary Figure 12: IP-pulldown for GFP-Rab11A cells during infection. An independent biological replicate of data shown in Figure 7 for the immunoprecipitation (IP) of GFP-Rab11A using anti-GFP magnetic beads. Infected cells showed slightly lower relative levels of GFP-Rab11A (0.91) compared to mock infected cells (1.0), while a much larger decrease in dynein motor proteins associated with GFP-Rab11A was observed (relative level = 0.38). Kif13A was not found to be associated with GFP-Rab11A for either the infected or mock cells even though it was detected in the input to the IP. Source data are provided as a Source Data file.

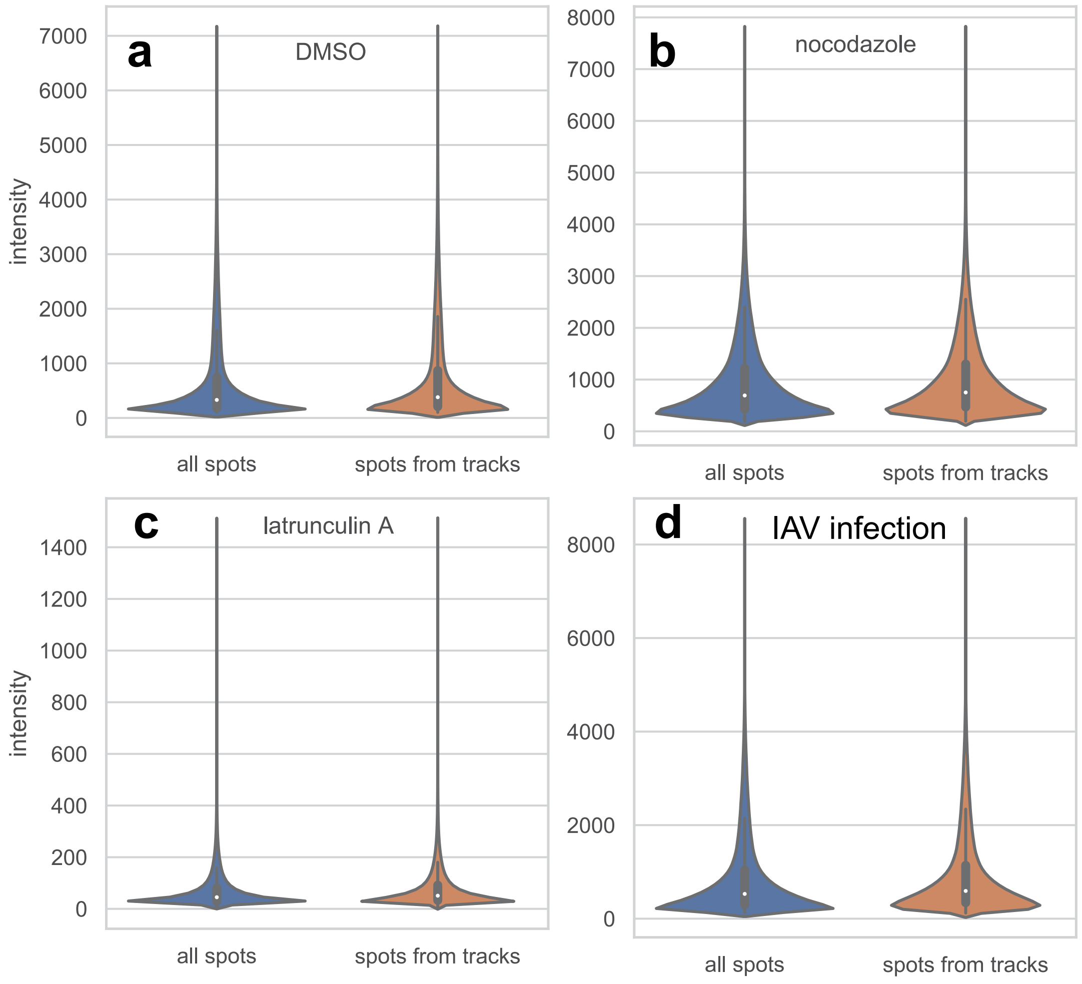

Supplementary Figure 13: Intensity distributions for spots identified during image analysis. As described in Materials and Methods, in the image analysis pipeline Rab11A spots are identified using Imaris and tracked for analysis. Only tracks longer than 3 consecutive timeframes are retained for statistical analysis. The intensity distribution of all identified spots are compared with the subset that are retained for tracking using violin plots (estimated kernel density distributions) in order to examine for any selection bias. Shown here are comparisons for Rab11A spots during **a**, DMSO treatment (n=14 cells), **b**, nocodazole treatment (n=6 cells), **c**, latrunculin A treatment (n=6 cells), and **d**, influenza A virus (IAV) A/WSN/33 infection (n=5 cells). Shown within the violin plots are median (white dot), inner quartiles (bold black lines), 1.5 times inter-quartile range (thin black lines). Plots made using the open-source Python's Seaborn package.
