# Supplementary material for: Quantitative live cell imaging reveals influenza virus manipulation of Rab11A transport through reduced dynein association
Source: Nat Commun. 2020 Jan 7;11:23. doi: 10.1038/s41467-019-13838-3 (PMC6946661; doi:10.1038/s41467-019-13838-3)
Supplement: Supplementary file 3 — Description of Additional Supplementary Files [file 41467_2019_13838_MOESM3_ESM.docx]

**Description of Additional Supplementary Files**

**File Name: Supplementary Movie 1**

**Description:** Image analysis workflow for spot identification and tracking. Sample dataset for live-imaging of A549 cells stably expressing GFP-Rab11A (white). For each time-lapse live-cell imaging dataset, the following steps are performed: define coverslip plane (white rectangle), define and select the cell volume (salmon color), define and crop the nuclear volume (in blue), identify spots (red) and create tracks (shown color-coded by time, blue is early time, red is late time) over the entire duration of the dataset.

**File Name: Supplementary Movie 2**

**Description:** Stalling, fusion and fission of GFP-Rab11A foci. A sample time-lapse movie of GFP-Rab11A foci traveling over a complex, interconnected cytoskeletal network. Two large stalled or tethered vesicles are highlighted by a white circle early in the movie. Multiple smaller vesicles fuse into and split from these stationary vesicles, which themselves slowly dissipate and later move away. Later in the movie, a white arrow identifies a moving vesicle that alternates between rapid and stalled motion at a cytoskeletal junction. Multiple fission and fusion events occur during the stalled phase after which the vesicle resumes rapid motion. The motion of such vesicles during slow or stalled periods is difficult to predict for tracking analysis.

**File Name: Supplementary Movie 3**

**Description:** A549 GFP-Rab11A cells under control treatment (DMSO). A549 cells stably expressing GFP-Rab11A were incubated with DMSO as control for 4 h and subsequently imaged. Rapid pervasive movement of Rab11A vesicles is observed with predominantly apical distribution.

**File Name: Supplementary Movie 4**

**Description:** A549 GFP-Rab11A cells under nocodazole treatment. A549 cells stably expressing GFP-Rab11A were incubated with microtubule depolymerizing drug nocodazole for 4 h and subsequently imaged. Rab11A vesicles are mislocalized to the basal periphery of the cells with highly diminished movement.

**File Name: Supplementary Movie 5**

**Description:** A549 GFP-Rab11A cells under latrunculin A treatment. A549 cells stably expressing GFP-Rab11A were incubated with actin depolymerizing drug latrunculin A for 4 h and subsequently imaged. The cellular morphology is highly disrupted under latrunculin A treatment, with the nucleus segregated to one side and the rest of the cell body to another. Robust Rab11A vesicle movement is observed.

**File Name: Supplementary Movie 6**

**Description:** A549 cells infected with H1N1 pandemic influenza expressing GFPtagged PA protein (control treatment). A549 cells were infected with H1N1pdm PA::GFP virus (MOI=1), treated with drug vehicle (DMSO) as control at 4 hpi and imaged at 16 hpi. Sample dataset shows fluorescent vRNP puncta undergoing cytoskeletal transport with multiple merging, temporary colocalization and splitting interaction events. The vRNP puncta are identified and tracked for statistical analysis.

**File Name: Supplementary Movie 7**

**Description:** A549 cells infected with H1N1 pandemic influenza expressing GFPtagged PA protein and treated with nocodazole. A549 cells were infected with H1N1pdm PA::GFP virus (MOI=1), treated with nocodazole at 4 hpi and imaged at 16 hpi. Sample dataset shows fluorescent vRNP puncta undergoing robust cytoskeletal transport with multiple merging, temporary colocalization and splitting interaction events. Compared to Rab11A motion under nocodazole treatment (Supplementary Movie 4), PA::GFP puncta maintain a significant portion of their movement. The vRNP puncta are identified and tracked for statistical analysis.

**File Name: Supplementary Movie 8**

**Description:** A549 cells infected with H1N1 pandemic influenza expressing GFPtagged PA protein and treated with latrunculin A. A549 cells were infected with H1N1pdm PA::GFP virus (MOI=1), treated with latrunculin A at 4 hpi and imaged at 16 hpi. Sample dataset shows fluorescent vRNP puncta undergoing robust cytoskeletal transport. The vRNP puncta are identified and tracked for statistical analysis.

**File Name: Supplementary Movie 9**

**Description:** A549 GFP-Rab11A cells infected with RFP-expressing RSV. A549 cells were infected with RSV (MOI=0.1 pfu/cell) and imaged at 16 hpi. Sample dataset shows fluorescent GFP-Rab11A puncta from a single cell that was volume cropped using RFP signal as a mask. This ensures that only GFP-Rab11A spots from RSV infected cells are analyzed. The vRNP puncta are identified and tracked for statistical analysis. In the movie, nuclear volume is highlighted (in blue) followed by all identified spots (red). Finally, only tracks with displacements greater 2 μm are shown along with their spots for clarity. Track colors correspond to relative time within the dataset, with blue being early and red being late timepoints.

**File Name: Supplementary Movie 10**

**Description:** A549 GFP-Rab11A cells infected with influenza virus B/Texas/02/2013. A549 GFP-Rab11A cells were infected with B/Texas/02/2013 (MOI=3) and imaged at 8 hpi. Sample dataset shows fluorescent GFP-Rab11A puncta undergoing robust cytoskeletal transport. The GFP-Rab11A puncta are identified and tracked for statistical analysis. In the movie, nuclear volume is highlighted (in blue) followed by all identified spots (red). Finally, only tracks with displacements greater 2 μm are shown along with their spots for clarity. Track colors correspond to relative time within the dataset, with blue being early and red being late timepoints.

**File Name: Supplementary Movie 11**

**Description:** A549 GFP-Rab11A cells infected with PA::mRuby virus. A549 GFPRab11A cells were infected with recombinant IAV H1N1 A/WSN/33 expressing mRuby-tagged PA. Each fluorophore was imaged sequentially at 16 hpi over 200 timepoints (timeframe interval 2.8 s). Sample dataset shows a cellular cross-section with signal from GFP-Rab11A puncta (green, left pane), PA::mRuby (magenta, middle pane) and their overlap (right pane). Fluorescent spots from both channels were identified and tracked for statistical analysis. Significant number of GFPRab11A and PA::mRuby spots are observed to collocate and co-travel, thus demonstrating their association during transport following an IAV infection.

**File Name: Supplementary Data 1**

**Description:** Processed data for mass spectrometry changes in detected protein quantities. Processed data for all detected proteins in mass spectrometry analysis for GFPRab11A for immunoprecipitation input as well as proteins pulled down by immunoprecipitation of GFP-Rab11A is shown. Included are protein descriptions, coverage, log2 fold change of the ratio of detected proteins in infected to mock-infected cells, and log2 of the p-value.

**File Name: Supplementary Data 2**

**Description:** Statistical tests results. This table includes degrees of freedom, confidence intervals, and p-values for figures 3-6 and supplementary figures 2, 4, 5, 8, 10, &11 presented in the manuscript.
