# Supplementary material for: Quantitative live cell imaging reveals influenza virus manipulation of Rab11A transport through reduced dynein association
Source: Nat Commun. 2020 Jan 7;11:23. doi: 10.1038/s41467-019-13838-3 (PMC6946661; doi:10.1038/s41467-019-13838-3)
Supplement: Supplementary file 17 — Reporting Summary [file 41467_2019_13838_MOESM17_ESM.pdf]

nature research

Corresponding author(s): Seema Lakdawala

Last updated by author(s): Nov 8, 2019

# Reporting Summary

Nature Research wishes to improve the reproducibility of the work that we publish. This form provides structure for consistency and transparency in reporting. For further information on Nature Research policies, see Authors & Referees and the Editorial Policy Checklist .

## Statistics

For all statistical analyses, confirm that the following items are present in the figure legend, table legend, main text, or Methods section.

| n/a | Confirmed | |
|---|---|---|
| ☐ | ☒ | The exact sample size (*n*) for each experimental group/condition, given as a discrete number and unit of measurement |
| ☐ | ☒ | A statement on whether measurements were taken from distinct samples or whether the same sample was measured repeatedly |
| ☐ | ☒ | The statistical test(s) used AND whether they are one- or two-sided<br>*Only common tests should be described solely by name; describe more complex techniques in the Methods section.* |
| ☒ | ☐ | A description of all covariates tested |
| ☐ | ☒ | A description of any assumptions or corrections, such as tests of normality and adjustment for multiple comparisons |
| ☐ | ☒ | A full description of the statistical parameters including central tendency (e.g. means) or other basic estimates (e.g. regression coefficient) AND variation (e.g. standard deviation) or associated estimates of uncertainty (e.g. confidence intervals) |
| ☐ | ☒ | For null hypothesis testing, the test statistic (e.g. *F*, *t*, *r*) with confidence intervals, effect sizes, degrees of freedom and *P* value noted<br>*Give P values as exact values whenever suitable.* |
| ☒ | ☐ | For Bayesian analysis, information on the choice of priors and Markov chain Monte Carlo settings |
| ☒ | ☐ | For hierarchical and complex designs, identification of the appropriate level for tests and full reporting of outcomes |
| ☒ | ☐ | Estimates of effect sizes (e.g. Cohen's *d*, Pearson's *r*), indicating how they were calculated |

*Our web collection on statistics for biologists contains articles on many of the points above.*

## Software and code

Policy information about availability of computer code

| | |
|---|---|
| Data collection | Commercial software: Olympus CellSense, Olympus Fluoview 1000<br>Custom software: Microscope controlling code written in LabView (code available on our Github page as mentioned in Materials and Methods) |
| Data analysis | The following commercial softwares were used: Imaris 8.4.2 (Oxford Instruments/Bitplane), Huygens version 18 (Scientific Volume Imaging), MATLAB R2018 (Mathworks), Graphpad Prism v7 and v8, Xcalibur software (Thermo Fisher Scientific), Proteome Discoverer (v2.3, Thermo Scientific), GeneGo's MetaCore pathways analysis package (Thomson Reuters) .<br>Open source software: ImageJ/FIJI, MIPAV (NIH)<br>Custom code: extensions to Imaris written in MATLAB (available on our GitHub), image registration and deconvolution code written in C++/CUDA (published at Biorxiv as mentioned in Materials and Methods, custom scripts written in Python/MATLAB (made available on our GitHub page as mentioned in the Materials and Methods) |

For manuscripts utilizing custom algorithms or software that are central to the research but not yet described in published literature, software must be made available to editors/reviewers. We strongly encourage code deposition in a community repository (e.g. GitHub). See the Nature Research guidelines for submitting code & software for further information.

## Data

Policy information about availability of data

All manuscripts must include a data availability statement. This statement should provide the following information, where applicable:

- Accession codes, unique identifiers, or web links for publicly available datasets
- A list of figures that have associated raw data
- A description of any restrictions on data availability

All data necessary for evaluating results are included in the manuscript. Data underlying Figures 3, 4, 5, 6, 7d, Supplementary Figures 2, 4, 5, 8, 10, 11, 12 are provided as Source Data files. Data underlying Supplementary Figure 1 is included as Supplementary Table 1. Data underlying Figure 7b, c are provided as

Supplementary Data 1 file. Raw Mass Spectrometry data are available via CHORUS project data repository (Project ID 1577). All of the raw diSPIM imaging data are available as open-source TIFF files on FigShare at [https://doi.org/10.35092/yhjc.c.4719353]. All statistical results including degrees of freedom, F values, p values and confidence interval information are provided as Suplementary Data 2 file. All other data are available from the corresponding author upon reasonable requests.

# Field-specific reporting

Please select the one below that is the best fit for your research. If you are not sure, read the appropriate sections before making your selection.

☒ Life sciences ☐ Behavioural & social sciences ☐ Ecological, evolutionary & environmental sciences

For a reference copy of the document with all sections, see nature.com/documents/nr-reporting-summary-flat.pdf

# Life sciences study design

All studies must disclose on these points even when the disclosure is negative.

| | |
|---|---|
| Sample size | The sample sizes were determined by the number of complete cell volumes that were acquired, that registered correctly and did not have technical problems such as signal intensity variations. Our sample sizes are sufficient because from within each cell, we identify and track hundreds of thousands of spots and tens of thousands of tracks. |
| Data exclusions | Some cells that looked very different from the rest of the sample were excluded. Unfortunately, because the microscope shows only a cross section at any given time, this is not apparent during imaging and the decision has to be made after registration and deconvolution. For the mass spectrometry one replicate was excluded out of three independent biological replicates because of poor pulldown efficiency. |
| Replication | Cells were imaged under multiple different conditions and treatments on multiple days. Data from cells acquired on multiple days have been pooled together. Effect of drugs and infections were replicated successfully from day to day. At least 4 different field of views were imaged for live-imaging experiments. |
| Randomization | No randomization was applied to the experiments on cells. |
| Blinding | Investigators were not blinded to the group allocation. Fairness was maintained by applying the same criterion for track generation distance thresholds and arrest coefficient thresholds across multiple treatment conditions in the analysis software. |

# Reporting for specific materials, systems and methods

We require information from authors about some types of materials, experimental systems and methods used in many studies. Here, indicate whether each material, system or method listed is relevant to your study. If you are not sure if a list item applies to your research, read the appropriate section before selecting a response.

## Materials & experimental systems

| n/a | Involved in the study |
|---|---|
| ☐ | ☒ Antibodies |
| ☐ | ☒ Eukaryotic cell lines |
| ☒ | ☐ Palaeontology |
| ☒ | ☐ Animals and other organisms |
| ☒ | ☐ Human research participants |
| ☒ | ☐ Clinical data |

## Methods

| n/a | Involved in the study |
|---|---|
| ☒ | ☐ ChIP-seq |
| ☒ | ☐ Flow cytometry |
| ☒ | ☐ MRI-based neuroimaging |

## Antibodies

| | |
|---|---|
| Antibodies used | All antibodies used in the study were commercially obtained and their manufacturer, part number and concentrations used are listed in the Materials and Methods section. |
| Validation | No validation was performed in our laboratory. All antibody validations are as listed on the supplier's website. |

## Eukaryotic cell lines

Policy information about cell lines

| | |
|---|---|
| Cell line source(s) | A549 human lung carcinoma cells, Madin-Darby Canine Kidney (MDCK) cells obtained from American Type Cell Culture |
| Authentication | Cell lines were not authenticated |

Mycoplasma contamination | Cell lines were not tested for mycoplasma contamination.

Commonly misidentified lines
(See ICLAC register) | *Name any commonly misidentified cell lines used in the study and provide a rationale for their use.*
